# Schizotypal traits in a large sample of high-school and university students from Tunisia: correlates and measurement invariance of the arabic schizotypal personality questionnaire across age and sex

**DOI:** 10.1186/s12888-023-04942-2

**Published:** 2023-06-20

**Authors:** Feten Fekih-Romdhane, Abir Hakiri, Manel Stambouli, Wissal Cherif, Rami Away, Amani Amri, Majda Cheour, Souheil Hallit

**Affiliations:** 1 The Tunisian Center of Early Intervention in Psychosis, Department of Psychiatry “Ibn Omrane”, Razi Hospital, 2010 Manouba, Tunisia; 2 Faculty of Medicine of Tunis, Tunis El Manar University, Tunis, Tunisia; 3 School of Medicine and Medical Sciences, Holy Spirit University of Kaslik, P.O. Box 446, Jounieh, Lebanon; 4 Psychology Department, College of Humanities, Effat University, Jeddah, 21478 Saudi Arabia; 5 Applied Science Research Center, Applied Science Private University, Amman, Jordan; 6 Research Department, Psychiatric Hospital of the Cross, Jal Eddib, Lebanon

**Keywords:** Schizotypy, Schizotypal traits, Adolescents and young adults, Schizotypal Personality Questionnaire, Measurement Invariance, Arabic

## Abstract

**Background:**

The main goal of the present study was to examine the characteristics of schizotypal traits and their correlations with genetic (i.e., family history of mental illness), demographic (i.e., age, sex), environmental (e.g., income, urbanicity, tobacco/alcohol/cannabis use), and psychological (i.e., personal history of mental illness other than psychosis) factors in Tunisian high-school and university students. Our secondary goal was to contribute the literature by examining the factor structure and factorial invariance of the Arabic Schizotypal Personality Questionnaire (SPQ) across sex and age (adolescents [12–18 years] vs. young adults [18–35 years]) groups.

**Method:**

This was a cross-sectional study involving 3166 students: 1160 (36.6%) high-school students (53.0% females, aged 14.9 ± 1.8); and 2006 (63.4%) university students (63.9% females, aged 21.8 ± 2.3). All students were asked to complete a paper-and-pencil self-administered questionnaire containing sociodemographic characteristics as well as the Arabic version of the SPQ.

**Results:**

The total sample yielded total SPQ scores of 24.1 ± 16.6 out of 74. The SPQ yielded good composite reliability as attested by McDonald's omega values ranging from .68 to .80 for all nine subscales. Confirmatory Factor Analysis indicated that fit of the 9-factor model of SPQ scores was acceptable. This model is invariant (at the configural, metric and structural levels) across sex and age. Except for “Odd or eccentric behavior”, all schizotypy features were significantly higher among female students compared to males. Multivariable analyses showed that female sex, being a university student, lowest family incomes, tobacco use, and having a personal history of psychiatric illness were significantly associated with higher positive, negative and disorganized schizotypy subscales scores.

**Conclusion:**

Future research still needs to confirm our findings and investigate the contribution of the identified factors in the development of clinical psychosis. We can also conclude that the Arabic SPQ is appropriate for measuring and comparing schizotypy across age and sex in clinical and research settings. These findings are highly relevant and essential for ensuring the clinical utility and applicability of the SPQ in cross-cultural research.

## Introduction

The interest is growing towards early stages of schizophrenia and the importance of their detection, given that some of these at risk mental states predisposing to schizophrenia may represent a target to many preventive and therapeutic interventions. However, this interest is not only focused on clinical population, as research has shown that the severity of psychotic symptoms seems to extend over a continuum of severity, ranging from subclinical psychotic manifestations in healthy subjects from the general population to psychotic disorders [1, 2]. From this perspective, schizotypy was described as a construct referring to the continuum of positive, negative and disorganized psychotic-like symptoms that range from the less pathological to the more pathological, being theoretically and empirically implicated in the prediction of schizophrenia spectrum disorders [3]. Indeed, there is sufficient evidence that high schizotypy can predict future onset of psychotic disorders [4, 5]. schizotypal traits resemble, phenomenologically and at subclinical level, the signs and symptoms of schizophrenia [6], which has been conceptualized as a latent liability for schizophrenia [7, 8]. Research focused on this last point stipulate that schizotypy, contingent on endogenous and exogenous factors, may ultimately lead to schizophrenia spectrum disorders [3, 8, 9]. For instance, Lenzenweger et al. [9] showed that elevated perceptual aberrations or schizotypal traits at age 18 predicted increased levels of psychotic symptoms and psychotic illness in midlife (17 years later), after adjusting for general non-specific psychopathology factors such as anxiety or depression. For these considerations, schizotypal features may constitute a useful endophenotype for genetic, neurobiological, and cognitive neuroscience investigations of schizophrenia liability [9].

In order to facilitate the study of the schizotypy entity, diagnostic criteria have been established, and questionnaires have been designed for the assessment of the different schizotypal dimensions. the schizotypal personality questionnaire (SPQ) is one of the most commonly used measures of schizotypy is [10] that has a clear connection to schizophrenia-associated biological vulnerability [11]. The SPQ is potentially useful as a screening instrument in the general population for clinical case formulation and early intervention efforts. Indeed, unlike most other self-report measures of schizotypy, the SPQ evaluates individual differences in nine schizotypal personality disorder (SPD) features according to the DSM criteria (i.e. excessive social anxiety, unusual perceptual experience, ideas of reference, odd speech, odd behavior, odd beliefs, constricted affect, no close friends, and suspiciousness) [10]. It is worth noting here that, given that the SPQ was modeled on SPD-symptoms, this could allow for capturing similar but not identical traits compared to other measures that have been developed with different conceptual backgrounds [12], such as the “Oxford-Liverpool Inventory of Feelings and Experiences” (O-LIFE, [13]). In the O-LIFE has been rather designed based on a dimensional schizotypy model that considers schizotypy as a source of healthy variation of personality throughout the population and not limited to the realm of illness [14]. As such, differences between the SPQ and other measures have been observed particularly regarding the nature of disorganized schizotypy [12]. The SPQ enables valuable information to be drawn on the main schizotypal factors, with one of the most widely used approaches being the subdivision of schizotypy into three dimensions (i.e. positive, negative and disorganized dimension) [10]. These different dimensions of schizotypy significantly interact during adolescence to sustain/exacerbate and perhaps increase the need for professional help in relation to symptom expression [15]. Each of the dimensions seem to have unique and distinct patterns of associations with schizophrenia-spectrum symptoms and functional impairment [16, 17]. In addition, schizotypal traits have been demonstrated to relate to a range of psychopathological indicators, including increased depressive and anxious symptoms [16, 17], attentional deficits [16, 18], substance use [19], technology addictions [20, 21], aggression [22], maladaptive coping strategies in life crises [23]. All these findings emphasize the high clinical relevance of the schizotypy construct in non-clinical individuals, above and beyond predicting later psychosis. Therefore, determining the phenotypic expression and different correlates of schizotypy in healthy sample derived from the general population is necessary for advancing our understanding of the origin, development, expression, and heterogeneity of schizophrenia; and may have potential clinical implications for prevention and early intervention of psychotic disorders.

Furthermore, the psychosis proneness-persistence-impairment model stipulates that schizotypy may interact additively or synergetically with other factors (i.e., genetic, sociodemographic, environmental, psychological) to precipitate the development of a full-blown psychosis [2, 24]. Indeed, it has been suggested that schizotypy shares the same demographic and environmental risk factors as those found in individuals with psychotic disorders, such as age, sex, marital status, urbanicity, income, family history of mental [7, 25]. Using schizotypy (a milder form of schizophrenia symptoms present in the general population [26]) as a model for researching schizophrenia might provide a useful approach to investigate sociodemographic differences in the disease symptomatology [27]; while not being affected by chronic and severe psychotic symptoms, cognitive impairment, and/or antipsychotics effects related to schizophrenia. There is empirical evidence suggesting that schizotypal manifestations differ according to certain sociodemographic characteristics, such as sex, age, and education [28–30]; though findings remain mixed. As for sex for example, previous findings tend to indicate that males in the general population tend to present more pronounced negative and disorganization schizotypal traits than females [30–34]. Other studies, however, showed inverted patterns of association of these dimensions with sex (e.g., [13, 35]). Prior research investigating sex differences in positive schizotypal traits have also yielded controversial findings, as there are observations of either males [33] or females [30, 32, 36] scoring higher in this dimension, and other observations finding no sex differences [13, 34, 37]. With regard to age, the findings are also inconclusive. Most of previous studies consistently reported negative correlations between positive schizotypal symptoms and age in the general adult population (e.g., [30, 35, 36, 38–41]), as well as positive correlations between age and negative traits [13, 36]. Nevertheless, other researchers did not find any significant correlations between schizotypy and age [42]. More studies are strongly needed to clarify some of these associations.

### Rationale and goals of the present study

Very little research attention has been directed toward the epidemiologic landscape of schizotypal traits in non-Western developing countries, with Arab countries being no exception. The lack of studies on this topic is partly explained by the fact that early intervention in psychosis is a new paradigm which has only very recently been introduced in the Arab world (in Tunisia more specifically). This major gap in the literature may hinder prevention and early intervention efforts in the Arab context, especially given that schizotypy and subthreshold psychotic symptoms are culturally-dependent constructs [43–45]. Interestingly, Wüsten et al. [46] found higher frequency of self-reported attenuated psychotic symptoms but less distress in people from low- and middle-income countries (LAMIC) than those from high-income countries. Khaled et al. [47] found that attenuated psychotic features are more commonly represented in Arabs than non-Arabs. More particularly, cross-national comparisons have shown that Tunisians exhibited the highest schizotypal traits scores compared to their counterparts from other parts of the world (including US, Canada, Europe, Australia, and China) [48]. This suggests that Tunisia, as an Arab North-African developing country, provides an appropriate context in which to investigate the multi-dimensional phenotypic continuum of schizotypy traits and experiences in individuals from the general population. We believe that providing descriptive data on the characteristics of schizotypal traits among people from different cultures has the potential to inform the scientific community about cultural differences in the broader psychosis-proneness phenotype that could be of high clinical value [49]. In this regard, the main goal of the present study was to investigate the characteristics and correlates of schizotypal features in a large sample of Tunisian students. More precisely, we aimed to examine the associations between schizotypal traits and genetic (i.e., family history of mental illness), demographic (i.e., age, sex), environmental (e.g., income, urbanicity, tobacco/alcohol/cannabis use), and psychological (i.e., personal history of mental illness other than psychosis) factors in adolescents and young adults.

Because schizotypal manifestations occurring early in life course are predictive of vulnerability to psychosis, psychometrically sound self-report instruments are pivotal for early detection of psychosis proneness from early adolescence to early adulthood. For this, we considered using the Arabic version of the SPQ [50], and expanding our target population for this study to include both high-school and university students, with a broad age range of participants (12–35 years). The SPQ has previously been validated in a sample of Tunisian university students aged 20.4 ± 1.4 years [50]; however, no evidence regarding its measurement invariance across sex groups were provided, and its validity in early adolescents remain unknown. To fill these gaps, our secondary goal was to examine psychometric properties of the Arabic SPQ in our sample before its use. We specifically aimed to examine its composite reliability, factor structure and factorial invariance across sex and age (adolescents [12–18 years] vs. young adults [18–35 years]) groups.

## Method

### Sample and procedure

A cross-sectional study was performed over 6 months, from November 2020 to April 2021. Participants consisted of high-school and university students drawn from 8 middle schools, 8 high schools and 3 universities in Tunis, Tunisia. A first contact with administrators was initially held to introduce the research project. A non-probability convenience sampling technique was used for the study. Students were approached in their classrooms after lectures; and invited by the researchers to participate if they: (1) were aged 12 to 35 years (because the ultrahigh-risk for psychosis population predominantly belongs to this age range [51]), and (2) had no personal history of psychosis or antipsychotic medication intake. A history of psychiatric illness (other than psychosis) was not an exclusion factor. Of the 3400 students approached to participate, 120 refused to participate and 114 were later excluded because they left some questions unanswered on the study questionnaires, resulting in a total final sample of 3166 students: 1160 (36.6%) high-school students (53.0% females, aged 14.9 ± 1.8 [Age range 12–18 years]); and 2006 (63.4%) university students (63.9% females, aged 21.8 ± 2.3 [Age range 18–35 years]).

### Ethics

Ethical approval was provided by the ethics committee of the Razi psychiatric hospital, Manouba, Tunisia. Approval to approach students was obtained from all directors and administrators of participating schools/universities. All students (aged > 18 years) gave their informed consent to participate. Also, each high-school student (aged < 18 years) provided a voluntary oral informed assent as well as a parental written informed consent before participation. Students’ parents who wished their child to participate were asked to sign the consent form and return it to the school. The study was performed following the Declaration of Helsinki for human research.

### Measures

All students were asked to complete a paper-and-pencil self-administered questionnaire divided into two parts. The first part contained demographic and psychosocial information: age, sex, living arrangement, monthly family income, residency, tobacco/alcohol/cannabis/other drugs use, as well as family and personal psychiatric history. The second part of the questionnaire contained the Schizotypal Personality Questionnaire (SPQ).

#### *The SPQ *[52, 53]

Students’ schizotypal traits were assessed using the SPQ. This instrument consists of 74 dichotomous items (Yes/No) and contains nine subscales for each symptom of the schizotypal trait: ideas of reference, odd beliefs and magical thinking, unusual perceptual experiences, suspiciousness, excessive social anxiety, no close friends, odd or eccentric behavior, constricted affect, and odd speech. In this study, we used the Arabic version of the SPQ [50], and the three-factor model: (1) *Negative factor* [no close friends (9 items), excessive social anxiety (8 items), suspiciousness (8 items), and constricted affects (8 items)], (2) *Positive factor* [odd beliefs and magical thinking (7 items), ideas of reference (9 items), suspiciousness (8 items), and unusual perceptual experiences (9 items)], and (3) *Disorganized factor* [odd speech (9 items), and odd or eccentric behavior (7 items)]. The SPQ revealed adequate psychometric properties in this study, with a Cronbach’s alpha for the SPQ subscales ranging from 0.89 to 0.91, and for the total SPQ of 0.90.

### Statistical analysis

#### Confirmatory factor analysis

We used data from the total sample to conduct a CFA via the SPSS AMOS v.29 software. The minimum sample size to conduct a CFA ranges from 3 to 20 times the number of the scale’s variables [54]. Therefore, we assumed a minimum sample of 1480 participants needed to have enough statistical power based on a ratio of 20 participants per one item of the scale, which was exceeded in our sample. Our intention was to test the original model of SPQ scores (i.e. 9-factor model). Values ≤ 5 for χ^2^/df, and ≤ 0.08 for RMSEA, and 0.90 for CFI and TLI indicate good fit of the model to the data [55]. However, these cut-off values should not be interpreted rigidly [56, 57]; values between 0.80 and 0.90 for CFI can indicate acceptable but mediocre fit to the data [58]. It should be noted that CFI and TLI are sensitive to the number of items [59].

#### Sex and age invariance

To examine sex and age invariance of SPQ scores, we conducted multi-group CFA [60] using the total sample. Measurement invariance was assessed at the configural, metric, and scalar levels [61]. Following the recommendations of Cheung and Rensvold (2002) [62] and Chen (2007) [60], we accepted ΔCFI ≤ 0.010 and ΔRMSEA ≤ 0.015 as evidence of invariance.

#### Further analyses

SPSS software version 23 was used to conduct data analysis. Composite reliability in both subsamples was assessed using McDonald’s ω, with values greater than 0.70 reflecting adequate composite reliability [63]. McDonald’s ω was selected as a measure of composite reliability because of known problems with the use of Cronbach’s α (e.g., [64]). The scores of first and second order were normally distributed, with their skewness and kurtosis varying between -1 and + 1 [65]. The Student t and ANOVA tests were used to compare two and three or more means respectively, whereas the Pearson correlation test was used to compare two continuous variables. Three linear regressions were conducted taking the three scores (negative factor, positive factor and disorganized factor) as a dependent variable respectively; variables that showed a *p* < 0.25 in the bivariate analysis were taken as independent ones. Variance Inflation Factor (VIF) values < 5 were used to check for the absence of multicollinearity [66]. *P* < 0.05 was considered significant at all times.

## Results

### Sample characteristics

**Fig. 1:**
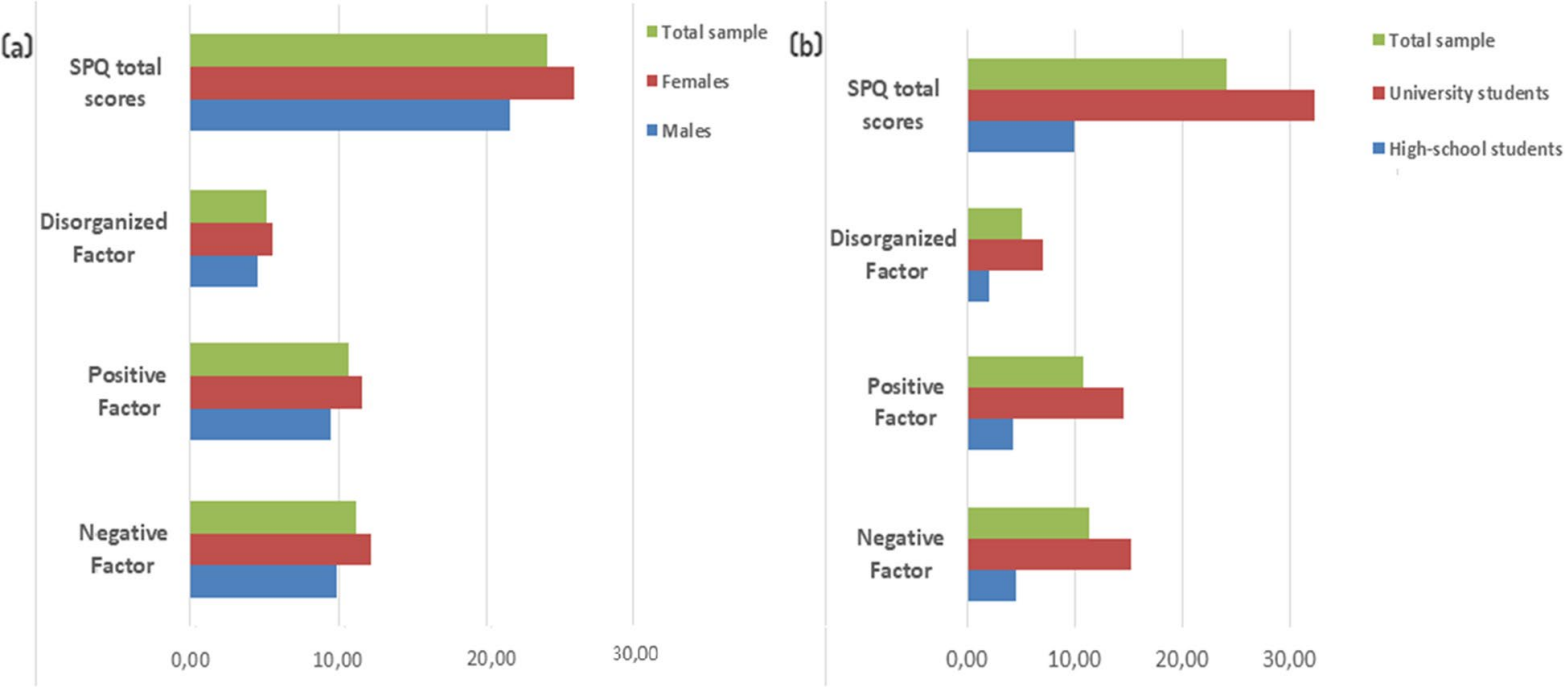
Total scores and sub-scores of the Schizotypal Personality Questionnaire by (a) sex and (b) population

A total of 3166 participants enrolled in this study; their mean age was 19.3 ± 3.9 years, with 59.9% females; 36.6% were adolescents and 63.4% were university students. Other details about the sample are summarized in Table 1. The total sample yielded total SPQ scores of 24.1 ± 16.6 out of 74 (Fig. 1).

**Table 1:** Sociodemographic and other characteristics of the participants

Variable, N (%)	Overall sample (*N* = 3166)	High-school students (*N* = 1160)	University students (*N* = 2006)
Sex			
Male	1269 (40.1%)	545 (47.0%)	724 (36.1%)
Female	1897 (59.9%)	615 (53.0%)	1282 (63.9%)
Habitation			
With parents	2395 (75.6%)	1157 (99.7%)	1238 (61.7%)
With friends	655 (20.7%)	3 (0.3%)	652 (32.5%)
Alone	116 (3.7%)	0	116 (5.8%)
Family income (in TD*)			
< 500	200 (6.3%)	39 (3.4%)	161 (8.1%)
500–1000	746 (23.6%)	266 (22.9%)	480 (23.9%)
1000–2000	1065 (33.6%)	416 (35.9%)	649 (32.3%)
2000–3000	700 (22.1%)	297 (23.0%)	403 (20.1%)
> 3000	455 (14.4%)	142 (12.2%)	313 (15.6%)
Residency			
Urban	2822 (89.1%)	1082 (93.3%)	1740 (86.7%)
Rural	344 (10.9%)	78 (6.7%)	266 (13.3%)
Tobacco use			
Yes	768 (24.3%)	149 (12.8%)	619 (30.8%)
No	2398 (75.7%)	1011 (87.1%)	1387 (69.1%)
Alcohol use			
Yes	713 (22.5%)	67 (5.8%)	646 (32.2%)
No	2453 (77.5%)	1093 (94.2%)	1360 (67.8%)
Cannabis use			
Yes	360 (11.4%)	69 (5.9%)	291 (14.5%)
No	2806 (88.6%)	1091 (94.0%)	1715 (85.5%)
Other illegal drug use			
Yes	124 (3.9%)	14 (1.2%)	110 (5.5%)
No	3042 (96.1%)	1146 (98.8%)	1896 (94.5%)
Personal psychiatric history other than psychosis			
Yes	885 (28.0%)	29 (2.5%)	856 (29.2%)
No	2281 (72.0%)	1131 (97.5%)	1150 (57.3%)
Family psychiatric history			
Yes	203 (6.4%)	42 (3.6%)	161 (8.0%)
No	2963 (93.6%)	1118 (96.4%)	1845 (92.0%)

#### Confirmatory Factor Analysis of the SPQ- First order

CFA indicated that fit of the 9-factor model of SPQ scores was acceptable: χ^2^/df = 12,791.30/2591 = 4.94, RMSEA = 0.035 (90% CI 0.034, 0.036), CFI = 0.845, TLI = 0.834. Standardized loading factors are summarized in Table 2.

**Table 2:** Items of the SPQ and Standardized Estimates of Factor Loadings from the Confirmatory Factor Analysis (CFA) in the total sample

**Factor 1 **Ideas of reference		**Factor 2 **Excessive social anxiety		**Factor 3 **Odd beliefs / magical thinking		**Factor 4 **Unusual perceptual experiences		**Factor 5 **Odd or eccentric behavior		**Factor 6 **No close friends		**Factor 7 **Odd speech		**Factor 8 **Constricted affect			**Factor 9** Suspiciousness	
Item	FL	Item	FL	Item	FL	Item	FL	Item	FL	Item	FL	Item	FL	Item	FL	Item		FL
1	.49	2	.50	3	.38	4	.51	5	.64	6	.50	7	.61	8	.59	9		.59
10	.49	11	.31	12	.59	13	.54	14	.60	15	.57	16	.55	17	.57	18		.57
19	.58	20	.47	21	.46	22	.41	23	.64	24	.58	25	.56	26	.31	27		.57
28	.62	29	.63	30	.48	31	.60	32	.53	33	.57	34	.64	35	.37	36		.59
37	.45	38	.55	39	.56	40	.48	67	.55	41	.47	42	.61	43	.58	44		.51
45	.44	46	.62	47	.42	48	.44	70	.54	49	.40	50	.56	51	.48	52		.62
53	.44	54	.61	55	.45	56	.46	74	.32	57	.61	58	.46	68	.37	59		.46
60	.57	71	.51			61	.52			62	.39	69	.50	73	.54	65		.58
63	.49					64	.57			66	.47	72	.49					

#### Composite reliability

Composite reliability of scores was adequate in the total sample for the subscales scores as follows: ideas of reference (ω = 0.76), excessive social anxiety (ω = 0.76), odd beliefs (ω = 0.68), unusual perceptual experiences (ω = 0.76), odd eccentric behavior (ω = 0.75), no close friends (ω = 0.76), odd speech (ω = 0.80), constricted affect (ω = 0.71) and suspiciousness (ω = 0.79).

#### Measurement invariance

Measurement invariance was supported accross sex and age as shown in Table 3. Higher mean ideas of reference, excessive social anxiety, odd beliefs / magical thinking, unusual perceptual experiences, no close friends, odd speech, constricted affect and suspiciousness scores were found in females compared to males. Moreover, adults scores higher on all scores compared to adolescents (Table 4).

**Table 3:** Measurement Invariance in the total sample

Model	χ^2^	*df*	CFI	RMSEA	Model Comparison	Δχ^2^	ΔCFI	ΔRMSEA	Δ*df*	*p*
Model 1: Invariance by Sex										
Configural	16,328.73	5182	.831	.026						
Metric	16,453.60	5247	.830	.026	Configural vs metric	124.87	.001	< .001	65	< .001
Scalar	16,689.36	5312	.828	.026	Metric vs scalar	235.76	.002	< .001	65	< .001
Model 2: Invariance by age										
Configural	24,240.59	5185	.634	.034						
Metric	24,592.93	5250	.628	.034	Configural vs metric	352.34	.006	< .001	65	< .001
Scalar	25,339.50	5312	.615	.035	Metric vs scalar	746.57	.013	.001	62	< .001

*Note.* *CFI* Comparative fit index, *RMSEA* Steiger-Lind root mean square error of approximation, *SRMR* Standardised root mean square residual

**Table 4:** *Differences in subscales scores between* sex *and age groups (N* = *3166)*

	**Sex**			**Age**		
	**Males**	**Females**	***p***	**Adolescents (12–18 years)**	**Adults (18–35 years)**	***p***
Ideas of reference	2.51 ± 2.31	3.09 ± 2.49	** < 0.001**	1.12 ± 1.40	3.75 ± 2.37	** < 0.001**
Excessive social anxiety	2.15 ± 2.04	2.84 ± 2.31	** < 0.001**	1.10 ± 1.23	3.31 ± 2.26	** < 0.001**
Odd beliefs / magical thinking	1.75 ± 1.76	2.10 ± 1.83	** < 0.001**	.80 ± 1.04	2.55 ± 1.83	** < 0.001**
Unusual perceptual experiences	2.47 ± 2.28	2.97 ± 2.43	** < 0.001**	1.18 ± 1.40	3.58 ± 2.38	** < 0.001**
Odd or eccentric behavior	2.03 ± 1.98	2.12 ± 2.05	0.241	1.00 ± 1.11	2.64 ± 2.16	** < 0.001**
No close friends	2.62 ± 2.33	3.14 ± 2.48	** < 0.001**	1.16 ± 1.38	3.84 ± 2.35	** < 0.001**
Odd speech	2.84 ± 2.48	3.47 ± 2.74	** < 0.001**	1.25 ± 1.39	4.23 ± 2.59	** < 0.001**
Constricted affect	2.36 ± 2.03	2.76 ± 2.12	** < 0.001**	1.16 ± 1.30	3.34 ± 2.04	** < 0.001**
Suspiciousness	2.78 ± 2.35	3.37 ± 2.45	** < 0.001**	1.14 ± 1.37	4.16 ± 2.21	** < 0.001**

#### Bivariate analysis

Higher mean negative, positive and disorganized factors values were seen in females compared to males, in university students compared to high school ones, in those who live with their friends or alone compared to living with their parents, in those who have a family income < 500 TD compared to the other categories, in those living in rural areas compared to urban, in those who use tobacco, alcohol, cannabis, other illegal drugs, in those who have a personal or family history or psychiatric illnesses (Table 5).

**Table 5:** *Bivariate analysis of factors associated with the negative, positive and disorganized factors scores (N* = *3166)*

Variable	Negative factor		Positive factor		Disorganized factor	
	**Mean ± SD**	***p***	**Mean ± SD**	***P***	**Mean ± SD**	***p***
Sex		** < 0.001**		** < 0.001**		** < 0.001**
Male	9.90 ± 7.58		9.51 ± 7.49		4.59 ± 3.71	
Female	12.11 ± 8.14		11.51 ± 7.91		5.57 ± 4.00	
Population		** < 0.001**		** < 0.001**		** < 0.001**
High school	4.57 ± 4.18		4.26 ± 4.38		2.04 ± 2.09	
University	15.08 ± 7.09		14.44 ± 6.86		6.99 ± 3.56	
Habitation		** < 0.001**		** < 0.001**		** < 0.001**
With parents	10.03 ± 7.96		9.54 ± 7.82		4.60 ± 3.89	
With friends	14.95 ± 6.87		14.26 ± 6.47		7.00 ± 3.39	
Alone	14.92 ± 6.93		14.69 ± 6.86		6.80 ± 3.51	
Family income (in TD*)		** < 0.001**		** < 0.001**		** < 0.001**
< 500	14.66 ± 7.74		13.55 ± 7.63		6.35 ± 3.80	
500–1000	12.06 ± 8.11		11.52 ± 7.83		5.55 ± 3.88	
1000–2000	10.92 ± 7.97		10.45 ± 7.86		5.08 ± 3.98	
2000–3000	10.40 ± 7.97		9.72 ± 7.67		4.78 ± 3.86	
> 3000	10.35 ± 7.49		10.25 ± 7.51		4.89 ± 3.79	
Residency		** < 0.001**		** < 0.001**		** < 0.001**
Urban	10.98 ± 7.93		10.52 ± 7.80		5.08 ± 3.91	
Rural	13.22 ± 8.28		12.22 ± 7.67		5.95 ± 3.83	
Tobacco use		** < 0.001**		** < 0.001**		** < 0.001**
No	10.55 ± 7.97		9.92 ± 7.70		4.78 ± 3.83	
Yes	13.35 ± 7.70		13.16 ± 7.61		6.42 ± 3.91	
Alcohol use		** < 0.001**		** < 0.001**		** < 0.001**
No	10.39 ± 7.96		9.80 ± 7.72		4.72 ± 3.84	
Yes	14.11 ± 7.43		13.82 ± 7.27		6.76 ± 3.74	
Cannabis use		** < 0.001**		** < 0.001**		** < 0.001**
No	10.91 ± 7.99		10.35 ± 7.78		5.00 ± 3.91	
Yes	13.67 ± 7.64		13.51 ± 7.41		6.53 ± 3.65	
Other illegal drug use		** < 0.001**		** < 0.001**		** < 0.001**
No	11.09 ± 7.99		10.57 ± 7.80		5.10 ± 3.91	
Yes	14.47 ± 7.29		14.10 ± 7.18		7.02 ± 3.60	
Personal psychiatric history other than psychosis		** < 0.001**		** < 0.001**		** < 0.001**
No	9.40 ± 7.53		8.72 ± 7.26		4.27 ± 3.67	
Yes	15.94 ± 7.17		15.82 ± 6.76		7.51 ± 3.51	
Family psychiatric history		** < 0.001**		** < 0.001**		** < 0.001**
No	11.02 ± 7.96		10.49 ± 7.73		5.09 ± 3.89	
Yes	14.26 ± 7.92		13.79 ± 8.19		6.50 ± 3.97	

^*^TD Tunisian Dinar, Numbers in bold indicate significant p-values

#### Multivariable analysis

Being female (Beta = 1.08), university students (Beta = 9.50), having a personal (Beta = 1.84) or a family (Beta = 1.18) history of psychiatric illness were significantly associated with higher negative factor scores, whereas having a family income between 500–1000 TD (Beta = -1.07), 1000–2000 (Beta = -1.74), 2000–3000 (Beta = -1.97) and > 3000 (Beta = 3.20) was significantly associated with lower negative factor scores (Table 6, Model 1).

**Table 6:** *Multivariable analyses (N* = *3166)*

	**Unstandardized Beta**	**Standardized Beta**	**p**	**95% CI**	**VIF**
**Model 1: Linear regression taking the negative factor score as the dependent variable**					
Sex (females vs males*)	1.08	0.07	** < 0.001**	0.63; 1.54	1.12
Population (university vs high school*)	9.50	0.57	** < 0.001**	8.95; 10.05	1.58
Habitation (with friends vs parents*)	-0.33	-0.02	0.269	-0.91; 0.26	1.26
Habitation (alone vs parents*)	-0.01	0.001	0.983	-1.18; 1.15	1.07
Family income (500–1000 TD vs < 500*)	-1.07	-0.06	**0.029**	-2.02; -0.11	3.70
Family income (1000–2000 TD vs < 500*)	-1.74	-0.10	** < 0.001**	-2.68; -0.80	4.42
Family income (2000–3000 TD vs < 500*)	-1.97	-0.10	** < 0.001**	-2.95; -0.99	3.73
Family income (> 3000 TD vs < 500*)	-3.20	-0.14	** < 0.001**	-4.24; -2.17	2.97
Residency (rural vs urban*)	0.18	0.01	0.618	-0.53; 0.90	1.11
Tobacco use (yes vs no*)	0.50	0.03	0.119	-0.13; 1.13	1.63
Alcohol use (yes vs no*)	-0.06	-0.003	0.858	-0.76; 0.63	1.88
Cannabis use (yes vs no*)	0.57	0.02	0.162	-0.23; 1.36	1.43
Other illegal drug use (yes vs no*)	0.41	0.01	0.507	-0.80; 1.62	1.23
Personal psychiatric history other than psychosis (yes vs no*)	1.84	0.10	** < 0.001**	1.32; 2.37	1.25
Family history of psychiatric illness (yes vs no*)	1.18	0.04	**0.008**	0.31; 2.06	1.03
**Model 2: Linear regression taking the positive factor score as the dependent variable**					
Sex (females vs males*)	0.97	0.06	** < 0.001**	0.53; 1.42	1.12
Population (university vs high school*)	8.77	0.54	** < 0.001**	8.23; 9.30	1.58
Habitation (with friends vs parents*)	-0.32	-0.02	0.271	-0.89; 0.25	1.26
Habitation (alone vs parents*)	0.29	0.01	0.617	-0.84; 1.42	1.07
Family income (500–1000 TD vs < 500*)	-0.66	-0.04	0.163	-1.59; 0.27	3.70
Family income (1000–2000 TD vs < 500*)	-1.31	-0.08	**0.005**	-2.22; -0.40	4.42
Family income (2000–3000 TD vs < 500*)	-1.81	-0.10	** < 0.001**	-2.77; -0.86	3.73
Family income (> 3000 TD vs < 500*)	-2.42	-0.11	** < 0.001**	-3.43; -1.41	2.97
Residency (rural vs urban*)	-0.24	-0.01	0.503	-0.93; 0.46	1.11
Tobacco use (yes vs no*)	0.92	0.05	0.003	0.31; 1.53	1.63
Alcohol use (yes vs no*)	0.003	0.001	0.994	-0.67; 0.68	1.88
Cannabis use (yes vs no*)	0.75	0.03	0.059	-0.03; 1.52	1.43
Other illegal drug use (yes vs no*)	0.14	0.003	0.815	-1.03; 1.31	1.23
Personal psychiatric history other than psychosis (yes vs no*)	2.67	0.15	** < 0.001**	2.16; 3.19	1.25
Family history of psychiatric illness (yes vs no*)	1.09	0.03	**0.012**	0.24; 1.94	1.03
**Model 3: Linear regression taking the disorganized factor score as the dependent variable**					
Sex (females vs males*)	0.54	0.07	** < 0.001**	0.31; 0.77	1.12
Population (university vs high school*)	4.30	0.53	** < 0.001**	4.03; 4.58	1.58
Habitation (with friends vs parents*)	-0.02	-0.002	0.910	-0.31; 0.28	1.26
Habitation (alone vs parents*)	-0.15	-0.01	0.610	-0.74; 0.43	1.07
Family income (500–1000 TD vs < 500*)	-0.07	-0.01	0.761	-0.56; 0.41	3.70
Family income (1000–2000 TD vs < 500*)	-0.35	-0.04	0.146	-0.82; 0.12	4.42
Family income (2000–3000 TD vs < 500*)	-0.53	-0.06	**0.035**	-1.02; -0.04	3.73
Family income (> 3000 TD vs < 500*)	-0.98	-0.09	** < 0.001**	-1.50; -0.46	2.97
Residency (rural vs urban*)	-0.05	-0.004	0.800	-0.41; 0.31	1.11
Tobacco use (yes vs no*)	0.50	0.05	**0.002**	0.18; 0.81	1.63
Alcohol use (yes vs no*)	0.12	0.01	0.514	-0.23; 0.46	1.88
Cannabis use (yes vs no*)	0.26	0.02	0.205	-0.14; 0.66	1.43
Other illegal drug use (yes vs no*)	0.30	0.02	0.330	-0.30; 0.91	1.23
Personal psychiatric history other than psychosis (yes vs no*)	1.05	0.12	** < 0.001**	0.79; 1.31	1.25
Family history of psychiatric illness (yes vs no*)	0.36	0.02	0.109	-0.08; 0.80	1.03

Nagelkerke R^2^ values: model 1 = 42.9%; model 2 = 43.3%; model 3 = 40.0%

Being female (Beta = 0.97), university students (Beta = 8.77), smoking (Beta = 0.92), having a personal (Beta = 2.67) or a family (Beta = 1.09) history of psychiatric illness were significantly associated with higher positive factor scores, whereas having a family income between 1000–2000 TD (Beta = -1.31), 2000–3000 (Beta = -1.81) and > 3000 (Beta = -2.42) was significantly associated with lower positive factor scores (Table 6, Model 2).

Being female (Beta = 0.54), university students (Beta = 4.30), smoking (Beta = 0.50), and having a personal history of psychiatric illness (Beta = 1.05) were significantly associated with higher disorganized factor scores, whereas having a family income between 2000–3000 TD (Beta = -0.53) and > 3000 (Beta = -0.98) was significantly associated with lower disorganized factor scores (Table 6, Model 3).

## Discussion

While psychosis features have been shown to vary widely across cultures, the vast amount of research on schizotypy and psychosis proneness has been restricted to Western countries [48]. To advance our knowledge of the complex profile of schizotypal traits and the extended psychosis phenotype across cultures, a study of the different characteristics of schizotypal traits and its correlates in community individuals from under-researched regions and countries are strongly needed. To this end, we investigated the prevalence and correlates of schizotypal traits in a large sample of Tunisian high-school and university students. Our main findings revealed that, except for “Odd or eccentric behavior”, all schizotypal features were significantly higher among female students compared to males. Multivariable analyses showed that female sex, being a university student, tobacco use, and having a personal history of psychiatric illness were significantly associated with higher positive, negative and disorganized schizotypal subscales scores; while having a higher family income was significantly associated with lower scores in all these three schizotypal dimensions. As for our secondary goal, our findings further confirmed that the Arabic SPQ has good psychometric qualities for measuring schizotypal trait expression during adolescence and young adulthood in non-clinical settings.

### Validation of the Arabic SPQ in early/late adolescents and young adults

A valid and reliable self-report measure is crucial to capture schizotypal traits in different age groups and both sexes. Particularly, it is recommended that schizotypal traits measures be specifically validated for adolescents before their use in this population [7]. Our findings revealed that the SPQ yielded good composite reliability as attested by McDonald's omega values ranging from 0.68 to 0.80 for all nine subscales. The factorial structure of the original first-order seven-factor model was adequately replicated. In addition, CFA showed that this model is invariant (at the configural, metric and structural levels) across sex and age. These findings are consistent with those of previous studies [30, 35, 36, 67, 68]. This suggests that the factorial structure underlying the SPQ in the adolescent group seem to be phenotypically similar to that found in the young adult group; which further supports the multidimensional nature of the schizotypal traits and suggests its stability during lifespan. It is of note, however, that although short-term test–retest reliability has proven appropriate in some studies (e.g., [69, 70]), others have shown that long-term test–retest reliability (e.g., over 2-years [51], and over 6 years [71]) is rather low. Some instability in schizotypal traits has also been evidenced in previous literature, with 75%–90% of subclinical psychotic manifestations in the general population seeming to disappear over time [2]. This has been proposed to be mainly due to plasticity and developmental changes that occur in childhood and adolescence; However, Raine et al. [72] provided evidence that counter these assumptions by showing stability of schizotypal traits even in childhood. We are aware, therefore, that future longitudinal studies are still required to attest for the stability of schizotypal traits over years; and further explore the reasons why schizotypal symptoms would change over time [72]. Finally, providing evidence of scalar, metric, and configural invariance enables researchers to reliably and confidently compare the means of the schizotypy construct between sex and age groups.

### Correlates of schizotypal traits in our sample

Regarding sex differences in schizotypal traits and symptoms, our findings showed that females displayed significantly greater scores in all schizotypy dimensions (positive, negative and disorganized), above and beyond other study variables. Data from previous literature was relatively limited [49] and has led to ambiguous findings. It has generally been documented that females tend to have higher levels of positive schizotypy and males tend to have higher levels of negative schizotypy [25, 31, 34, 40, 73]. Some observations suggest that females seem to report higher disorganized schizotypy scores [13, 74], while others concluded that male subjects had a greater expression of the disorganized factor score [30, 32, 40]. A meta-analysis pooled 44 studies (including 41 003 participants from 12 Western/Eastern countries) on sex differences in schizotypy, and demonstrated that men scored higher on the scales of negative schizotypal features; while no significant sex differences were found in the measures of positive schizotypal features [34]. This meta-analysis also showed that sex differences in Perceptual Aberration were larger in studies with nonstudent and older samples [34], which suggests that including only students may partly explain the previously documented inconsistencies regarding sex. Future research using nonstudent samples from the general population are needed to confirm our findings. Other explanations of the mixed previous findings can also be advanced. Our findings regarding correlations of schizotypal traits with sex may be partly explained by the measure used in our study. Indeed, unlike other measures, the SPQ has been found to be influenced by higher-order dimensions of personality (i.e., Neuroticism) [75]; which is, in turn, consistently found to be highly displayed by females [76]. Furthermore, there is evidence supporting that males and females may experience different responses to risk factors of psychosis, including sex-related sociocultural and biological processes [77]. For example, some authors explain these differences by the influence of confounding factors, such as greater alcohol and cannabis use in males [78], which often are not adjusted for in research [77]. However, tobacco, alcohol and cannabis use were all controlled for in our study, which denies this possibility. Other factors could be reported to a greater extent among females than males in the general population, such as mood symptoms [79]; which are also over-represented in highly schizotypal individuals [20, 21, 80]. It has also been suggested that schizotypy features do not correlate with psychopathology in the same way across sex [81]. Future studies should control for these factors when making sex comparisons across schizotypy features.

Overall, 63.4% of the sample were university students and scored higher on the positive, negative and disorganized factor scores. These findings are broadly consistent with previous meta-analytic evidence that the prevalence of attenuated psychotic symptoms are substantially higher among university students (25.40%) compared with their high-school counterparts (18.90%) [82]. It is of note, however, that when investigating the effect of age on schizotypal traits in non-clinical populations, most of the previous studies were restricted to adult samples [30, 40, 42]. For example, a study among Mexican adults aged 18 to 84 (mean age of 34.58 years) found that younger participants had higher scores on ideas of reference, excessive social anxiety, no close friends, and odd speech than those who were older [68]. Similar findings have been reported in a study among Turkish individuals aged 16–90 years (mean age of 30.5 years) [40]. As such, the generally accepted idea that age is inversely linked to subclinical expressions of psychosis (e.g., psychotic-like experiences, schizotypal trait) [25, 83] seem to be driven by comparisons between young adults and older adults. Future studies only focusing on the adolescent/young adult populations and using the same measurement tool may help clarify the previous non concluding findings regarding age and schizotypy.

Another important finding of this study was that lowest family incomes were associated with greater positive, negative and disorganized schizotypy scores. The largest amount of research on the association between socioeconomic status and psychosis focused on psychotic disorders [84–86]. Findings in this regard suggest that people with unfavorable economic conditions are more likely to experience social adversities [87, 88], encounter detrimental stressors and face barriers to resources, which place them at heightened risk for developing psychosis [89]. Evidence on the positive association between economic factors and attenuated psychosis has been recently supported in a study among US undergraduate and graduate students; where authors found that multiple socioeconomic factors (childhood, current, and pandemic-related financial stress and food insecurity) were increasingly related to increased odds of experiencing psychotic symptoms in a dose–response fashion [89].

Another factor that was strongly and significantly associated with positive and negative schizotypy scores in our study was the presence of a personal and/or family history of psychiatric illness. This result is consistent with previous evidence stipulating that schizotypal traits are more prevalent in individuals with a family history of schizophrenia or other psychotic spectrum diseases [90, 91]. There is substantial evidence that genetic liability to schizophrenia is present among non-psychotic relatives of schizophrenia patients [92], and can produce observable “schizophrenia-like” traits in these relatives even in the absence of frank psychosis [93]. Prior research has, for example, shown that social interpersonal schizotypal symptoms were more represented in individuals with a high family genetic load than those with no family psychiatric illness [93]. Another research found that a familial risk for developing schizophrenia was associated with negative schizotypy scores in adolescents [90]. An Italian study among 1023 high-school students showed that a family history of psychosis was significantly present in the “true schizotypal group” compared to the “unusual subjective experiences” group [91]. The results for disorganization symptoms are however inconclusive [93].

Finally, although we found higher means of positive, negative and disorganized scores in students who used tobacco, alcohol, cannabis, and other illegal drugs; these associations were no longer retained after the multivariate analysis, except for tobacco use which was significantly associated with the positive and the disorganization factor scores. Data concerning the link between cannabis use and attenuated psychosis in general, and schizotypal features in particular, remain mixed. Gina et al. [94] found that cannabis use and cannabis related problems were significantly higher in the highly schizotypal group. However, these results are still controversial. Another Spanish study involving 15,888 students found no significant associations between cannabis use and schizotypal traits [19]. On the other hand, this study revealed that alcohol users scored higher on social the disorganization schizotypy dimension; and that cigarette smokers reported higher average scores in the anhedonia and social disorganization dimensions than non-smokers [19]. A 10-year longitudinal study demonstrated that higher levels of positive schizotypal symptoms were associated with increased rates of alcohol use in non-clinical college students [95]. Esterberg et al. [96] found similar non-significant differences. Compton et al. [97] found higher schizotypy levels in early adults (aged 25–29) with heavier use of alcohol and cannabis. Contrarily, another study found that alcohol user students did not display higher scores on positive schizotypy, but had lower scores on negative schizotypy [98]. A lack of significant associations between alcohol use and schizotypal symptomatology has also been highlighted in other clinical and nonclinical samples (e.g., [99, 100]). More recently, Dinzeo and Thayasivam [101] found no significant associations between schizotypy features (symptoms’ presentation and severity) and substance use (alcohol, cannabis, amphetamines, cocaine, opioids, sedatives, hallucinogens) in US community young adults; albeit zero-order correlations suggested a link between nicotine use and disorganized schizotypy. These differences may be partly explained by wide variations in how substance use is assessed. Taking cannabis as example, categorizing students binary as lifetime users vs. non-users may not allow for distinguishing different frequency-of-use [102, 103] and/or potency (i.e., the concentration of THC in cannabis) [104] levels, which can be determinant for the risk of psychosis development [104–109].

### Clinical and research implications

Some preliminary implications can be drawn when considering the pattern of the present findings. A number of correlates of schizotypy have been identified in our population. Specifically, female sex, being a university student, nicotine use, low family income and having a family/personal history of psychiatric illness were associated with more severe schizotypy features. Unlike previous studies, female and older students scored higher in all three schizotypy dimensions (positive, negative and disorganized). The majority of previous research used data in adult samples of restricted [30, 42] or large [40, 68] age range; whereas we included students aged 12–35 years, because the at-risk for psychosis population predominantly belongs to this age range [110]. As such, one of the relatively new aspects of our study is the comparison of schizotypy severity between high-school and university student populations. Pending further scrutiny, we suggest that schizotypal manifestations seem to be less apparent in early adolescence, and gradually increase to manifest more clearly later in late adolescence and early adulthood.

In sum, the present findings support previous observations that like schizophrenia spectrum disorders, a range of genetic, demographic, environmental and psychological factors appear to underpin schizotypal traits and symptoms [111, 112], thus adding further evidence to the continuum model of psychosis. More research from underrepresented regions and countries are still needed to further identify correlates of schizotypal manifestations, and clarify the influence of these correlates on psychosis outcomes. Our study also supported measurement invariance of the Arabic SPQ scores, thus offering preliminary validity evidence for the factorial equivalence of schizotypal traits and symptoms across age and sex. These findings are highly relevant and essential for ensuring the clinical utility and applicability of the SPQ in cross-cultural research, as well as for advancing our knowledge of the complex phenotypic expression of schizotypal traits from a developmental perspective.

### Study limitations

The present findings need to be interpreted with considering the following limitations. First, because of the cross-sectional nature of the study, we were only able to report associations rather than conclude to definitive temporal or causal relationships. Second, we relied on a self-report measure to assess schizotypy, which may lead to overestimated prevalence of schizotypal symptoms compared with interviewer-based methods. It has been reported, for example, that self-report methods may be discordant with clinician ratings in some schizotypy features, including interpersonal and disorganization traits; and are less able to differentiate between non-clinical high-schizotypal individuals and those with Schizotypal personality disorder [113]. Future research should consider using both self-report and clinician ratings. Third, our analytic study was based on the three model factor of the SPQ; while some recent studies suggested that the three model factor may not be the best way to interpret the SPQ [114, 115]. Additional studies in our context are needed to improve the predictive value of the SPQ. Fourth, we adopted in our study a three-factor model that has previously provided a good fit to the data, accounting for 70.7% of the total variance of the scale in a sample of university students [50]. This model has also been considered in other Tunisian studies among university students (e.g., [20, 21, 54]). However, we could not perform the second order CFA using SPSS Amos since the same suspiciousness items load on 2 factors (negative factor and positive factor).

## Conclusion

This study is among the first to explore the nature and correlates of schizotypy dimensions in a large sample of adolescents and young adults recruited in non-clinical settings from an Arab developing country of the Middle East and North Africa (MENA) region, Tunisia. Findings preliminarily revealed that all schizotypy dimensions appear to be more evident in late than early adolescence, and to be more prominent in females than male students. Other correlates of schizotypy have been identified, namely tobacco use and personal/family history of psychiatric illness. To date, the etiology of psychosis is still unknown, and there seems to be a complex interplay between psychosis proneness and genetic, demographic, environmental, and psychological factors. Future research should confirm our findings and investigate the contribution of the identified factors in the development of clinical psychosis. Through the current study, we can also conclude that the Arabic version of the SPQ is appropriate for measuring and comparing schizotypy across age and sex in clinical and research settings.

## Data Availability

All data generated or analyzed during this study are not publicly available due the restrictions from the ethics committee. Reasonable requests can be addressed to the corresponding author.
